# Exploring the Impact of an Interactive Electronic Pegboard on Manual Dexterity and Cognitive Skills of Patients With Stroke: Preliminary Analysis

**DOI:** 10.2196/55481

**Published:** 2024-10-24

**Authors:** Shih-Ying Chien, Ching-Yi Wu, Alice May-Kuen Wong, Chih-Kuang Chen, Sara L Beckman

**Affiliations:** 1 Department of Industrial Design Chang Gung University Taoyuan Taiwan; 2 Department of Physical Medicine and Rehabilitation Chang Gung Memorial Hospital at Linkou Taoyuan Taiwan; 3 Department of Occupational Therapy Chang Gung University Taoyuan Taiwan; 4 Healthy Aging Research Center Chang Gung University Taoyuan Taiwan; 5 School of Medicine Chang Gung University Taoyuan Taiwan; 6 Center of Comprehensive Sports Medicine Chang Gung Memorial Hospital at Taoyuan Taoyuan Taiwan; 7 Haas School of Business University of California, Berkeley Berkeley, CA United States

**Keywords:** interactive electronic pegboard, stroke, hand dexterity, cognitive rehabilitation, system

## Abstract

**Background:**

As individuals age, the incidence and mortality rates of cerebrovascular accidents significantly rise, leading to fine motor impairments and cognitive deficits that impact daily life. In modern occupational therapy, assessing manual dexterity and cognitive functions typically involves observation of patients interacting with physical objects. However, this pen-and-paper method is not only time-consuming, relying heavily on therapist involvement, but also often inaccurate. Digital assessment methods, therefore, have the potential to increase the accuracy of diagnosis, as well as decrease the workload of health care professionals.

**Objective:**

This study examined the feasibility of an interactive electronic pegboard for the assessment and rehabilitation of patients with stroke.

**Methods:**

We explored the pegboard’s clinical applicability by examining the relationship among stages, timing, and difficulty settings, as well as their alignment with patient capabilities. In total, 10 participants used a prototype of the pegboard for functional and task assessments; questionnaire interviews were conducted simultaneously to collect user feedback.

**Results:**

Patients with stroke consistently required more time to complete tasks than expected, significantly deviating from the initial time frames. Additionally, the participants exhibited a slight reduction in performance levels in both manual dexterity and cognitive abilities. Insights from questionnaire responses revealed that the majority of participants found the prototype interface easy and enjoyable to use, with good functionality.

**Conclusions:**

This preliminary investigation supports the efficacy of interactive electronic pegboards for the rehabilitation of the hand functions of patients with stroke, as well as training their attentional and cognitive abilities. This digital technology could potentially alleviate the burden of health care workers, positioning it as a valuable and intelligent precision health care tool.

## Introduction

### Background

The population of older adults worldwide is likely to continue to increase in the coming decades [1,2]. Older adults face an array of economic, psychological, and societal challenges, as well as increased vulnerability to diverse chronic ailments [3-5]. Among these, hypertension is a critical concern closely associated with the occurrence of strokes [6,7]. In 2020, 7.08 million individuals succumbed to cerebrovascular disorders [8], and over 12.2 million people are diagnosed with new strokes each year. That means 1 in 4 individuals worldwide over the age of 25 years will experience a stroke in their lifetime [7]. Due to continuous advancements in medical technology, the survival rate for patients with stroke has reached approximately 62% [9]. However, there is an almost 90% probability that survivors of stroke will experience residual effects, which underscores the importance of rehabilitation and occupational therapy [10,11].

Cerebral stroke, arising from damage to cerebral tissue, induces varied neurological symptoms contingent upon the site of injury. These symptoms often give rise to impairments in motor function, sensory perception, and cognition, which can manifest as diminished attentional focus and memory deficits [12,13]. These changes in capabilities often entail significant consequences for patients, affecting their daily functioning, occupational status, and social engagement [14].

To enhance the physical mobility, manual skills, and cognitive abilities of patients with stroke, physical or occupational therapy is commonly used in clinical settings as a foundational approach [15-18]. Occupational or physical therapists frequently use toys, such as building blocks, to train patients in hand dexterity, hand-eye coordination, bilateral coordination, visual perception, and attention [19,20]. In line with this approach, the Nine-Hole Peg Test (NHPT) and the Purdue Pegboard Test (PPT) are commonly used for the assessment of manual dexterity [21]. Whether they are used for assessment or rehabilitation, these activities are routinely conducted in a 1-on-1 format, and both involve the use of a countdown timer to measure the time taken by patients to complete each task. This method has been substantiated in clinical settings as effective for the assessment of attention, cognition, and manual dexterity [22,23]. The commercially available Neofect Smart Pegboard is designed to enhance the training process by making it more interesting and interactive using audiovisual features. Upon completion of the training, the device calculates outcomes, such as accuracy and the time taken to place pegs. Despite the considerable advancements of the Neofect Smart Pegboard in aiding patients with stroke in rehabilitation and cognition, there remains significant room for improvement.

### Tools for Rehabilitation of Patients With Stroke

Pegboards are widely used in rehabilitation facilities to train patients in lateral coordination, manual dexterity, hand-eye coordination, as well as visual perception and attention. However, in the rehabilitation process, patients with stroke need to engage in active and prolonged exercises. The repetitive and high-intensity nature of these exercises can lead to boredom, fatigue, or laziness among patients, making it challenging for them to remain focused. Immediate feedback and interaction are both effective approaches to increasing user engagement.

**Figure 1:**
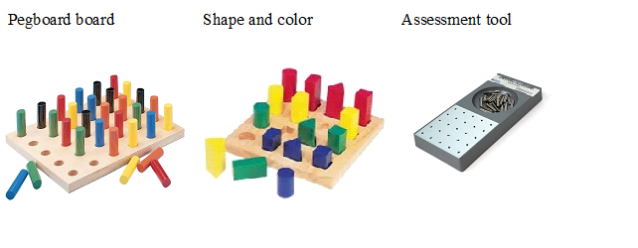
Traditional tools for manual dexterity and cognitive training and assessment.

Due to individual differences in the rehabilitation needs of patients with stroke, comprehensive rehabilitation records can assist physicians and therapists in designing personalized training programs. The use of traditional pegboards relies on manual documentation by health care professionals, which can be cumbersome and prone to errors in clinical settings. Furthermore, the majority of traditional training equipment cannot synchronize training, assessment, and testing for functions such as manual dexterity, visual perception, and attention. Without a means of integrating singular evaluations, there is a lack of objective assessment criteria (Figure 1). Although the Neofect Smart Pegboard reduces the costs of human resources, it fails to achieve individualized prescription and tracking functionalities.

### Study Objective

The primary objective was to devise personalized treatment plans that address the specific needs and goals of each patient. This systematic approach will contribute to enhancing patients’ capabilities in everyday life and professional endeavors. The challenges inherent to traditional assessment and training approaches, such as heightened time demands and difficulties in precisely monitoring disease progression or managing data entry errors, can be effectively alleviated through the integration of digital technology [24,25]. Thus, this study developed a prototype of an innovative interactive electronic pegboard for the rehabilitation of patients with stroke. By integrating electronic sensing technology and wireless network capabilities (ie, WiFi), the proposed system will enhance the precision and effectiveness of rehabilitation training and assessment. Digitalization of the process will facilitate accurate tracking of a patient’s progress and enable individualized assessments of movement and cognitive issues, thus promoting high-quality personalized health care [26,27].

## Methods

### System Design

We validated the feasibility of the proposed interactive electronic pegboard for clinical training and assessment through evaluation of its functionality and usability.

**Figure 2:**
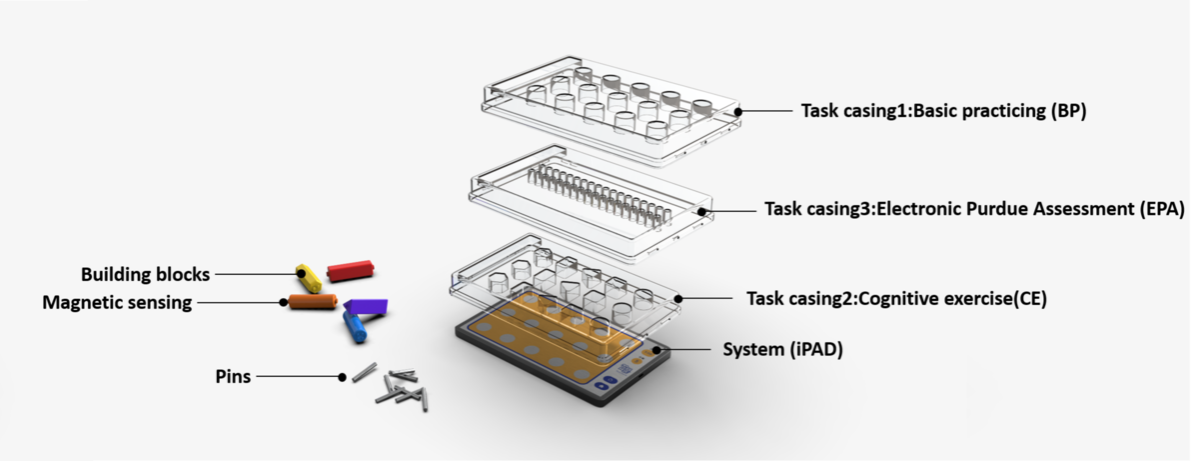
System design for the interactive electronic pegboard.

**Figure 3:**
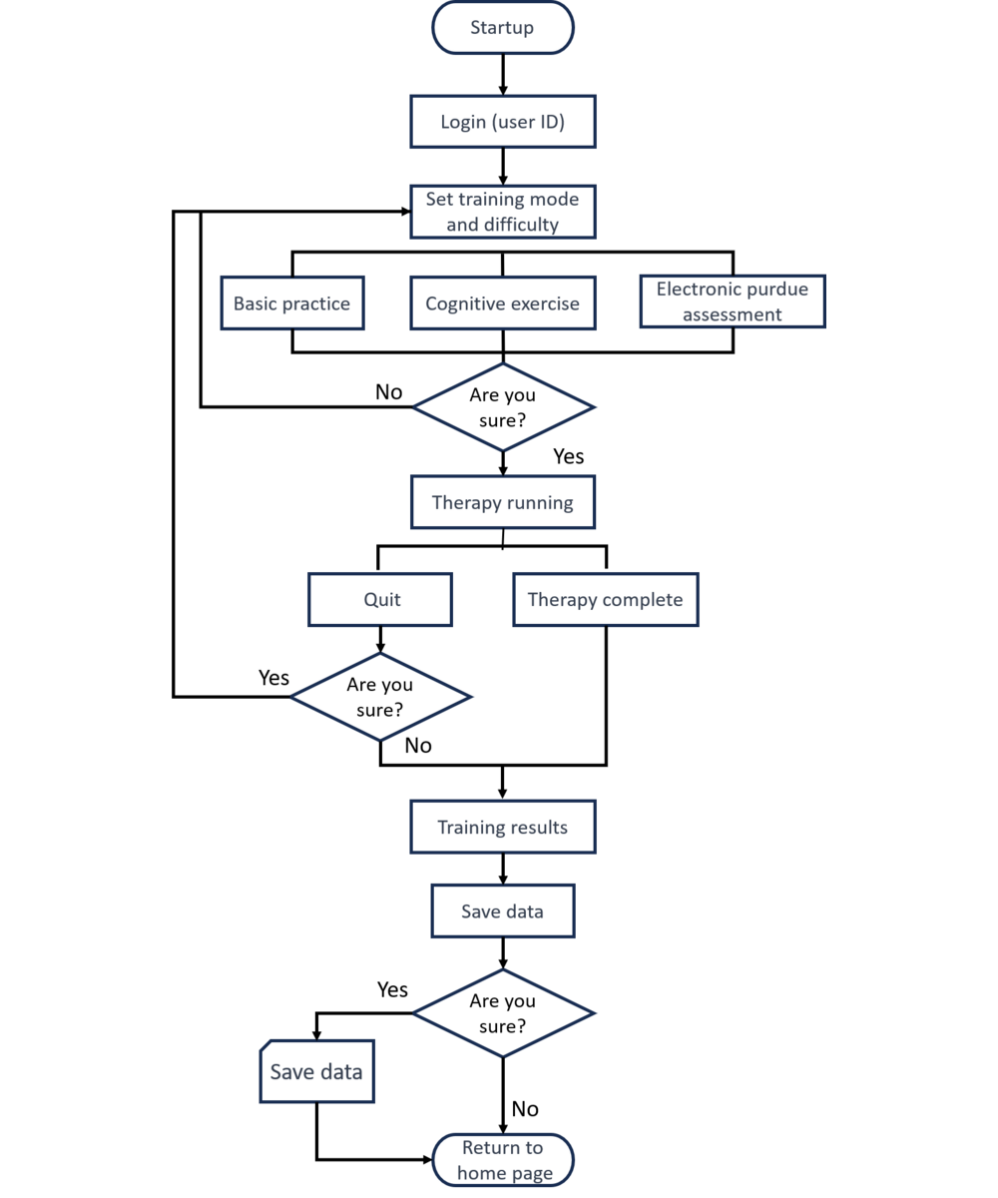
Interactive electronic pegboard system flowchart.

The proposed system comprises an iPad (Apple Inc), responsive building blocks, and 3 task casings (Figure 2). The building blocks used in the experiment are constructed from conductive materials (magnetic sensors). Analogous to the principles of touch panels, when these conductive building blocks come into contact with the panel, they facilitate communication with the iPad through capacitive touch interactions. The principle underlying color recognition uses the multitouch capability of the tablet. It relies on the distance between points and single- or multiple-touch conditions as the basis for color interpretation by the computer. Each building block has distinctive visual patterns on its rear surface. When a user places a building block onto the tablet, the tablet’s capacitive touch technology detects the applied force area, which is then used to determine the color of the block. A flowchart of the interactive electronic pegboard’s system is depicted in Figure 3.

The system comprises 3 modes: basic practice (BP; motor training), cognitive exercise (CE; shape and color matching), and Electronic Purdue Assessment (EPA). Each mode includes a range of difficulty levels. All 3 modes can be configured as timed or untimed. In the timed mode, users must complete tasks within the designated time, and algorithmic exercises and test evaluations are conducted based on the achieved results. Relevant reports are accessible to both therapists and patients, and all data are automatically stored in the cloud. Next, we describe the 3 modes and their difficulty levels in detail.

#### Basic Practice

**Figure 4:**
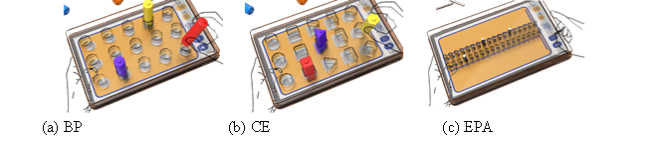
Three modes of the proposed interactive electronic pegboard task. BP: basic practice; CE: cognitive exercise; EPA: Electronic Purdue Assessment.

In this mode, patients are required to match signals displayed on the tablet screen with corresponding building blocks, as illustrated in Figure 4a. At the basic level, patients are only asked to place blocks in their designated positions. The intermediate level asks for precise alignment of both color and position. The advanced level incorporates a speed variable to augment the complexity of the task.

#### Cognitive Exercises

In this mode, patients engage in the assessment of cognitive concepts. Similar to the BP mode, they are required to match building blocks to the pattern presented on the screen to earn points. However, additional cognitive challenges are introduced, such as not only placing the blocks in the correct positions but also considering their shape and color, as shown in Figure 4b. In advanced levels, a speed variable is introduced to heighten the complexity of the task.

#### Electronic Purdue Assessment

This mode was designed in alignment with the principles of the PPT, where illuminated signals guide patients in the sequential insertion of pegs into corresponding holes. This assessment comprises distinct 30-second trials for the left hand, the right hand, and both hands. The platform automatically calculates the average completion time over 3 trials (see Figure 4c).

### System Log-on and Personal Record and Prescription

Big data analytics enables the system to efficiently estimate personalized rehabilitation prescriptions. To accomplish this, the system incorporates a user log-on mechanism for the effective management of individual records. The 3 modes include preset exercises encompassing a variety of directional movements. By analyzing repeated user interaction records, machine learning estimates individual needs and prescriptions. It focuses on repetitive exercises tailored to address the weaker areas of each patient.

### Data Collection

To assess whether this design can meet user needs for product functionality, this study adopted the happiness, adoption, and task success metrics from Google’s user experience framework as indicators to examine users’ subjective perceptions of the prototype. These metrics included satisfaction (happiness); acceptance (adoption), such as interest in the prototype; and task success, which represents the users’ success rate in accomplishing specific tasks within the product. Accordingly, these metrics served as a means to collect emotional experiential information from users during operation.

To validate the benefits of the proposed system, we collected the following data:

- Discrepancies between the time and difficulty settings of the proposed system and the patients’ capabilities
- Correlation between the scores calculated by the proposed system and therapist evaluations of hand function/cognitive abilities to determine the accuracy of the system
- User feedback

### Participants

The pilot study was conducted at the Department of Physical Medicine and Rehabilitation, Chang Gung Hospital, Taiwan. Participants were recruited through purposive sampling, as recommended by rehabilitation specialists. The inclusion criteria required adults aged 55 years or older who had been diagnosed by a physician and were undergoing rehabilitation following a mild stroke. In total, 10 older adults were enrolled. All participants were right-handed, fully conscious, able to communicate their sensory experiences in Mandarin or Taiwanese, and provided informed consent after the study procedures were explained. Exclusion criteria included individuals at risk for a secondary stroke, those with cognitive impairments that prevented verbal communication or cooperation, and individuals with severe mental or cognitive disorders.

### Ethical Considerations

The research protocol underwent rigorous scrutiny and received approval from the Research Ethics Board of Chang Gung Hospital (reference number IRB/REC 202301197A3). Prior to participation, each participant provided informed consent by signing the necessary documentation and was informed that the trial was conducted on a voluntary basis, allowing them to withdraw at any time without affecting their rights to treatment. The data obtained from this trial will be used solely for research purposes and not for any other application. All case information and data have been anonymized, with only numerical codes used for identification during the analysis to ensure privacy. Once the collected data were individually encoded and anonymized, the information was entered into a computer and processed using SPSS version 20.0 (IBM Corp) for statistical analysis.

### Experiments

This pilot study primarily focused on digitizing rehabilitation records using BP and CE modes as specific test components. The aim was to evaluate the differences between the time, operating mode, and difficulty settings of the proposed system and the capabilities of the patients. Furthermore, we aimed to confirm the efficacy of the system by assessing the correlation between color and shape in evaluations of hand function and cognitive abilities. Finally, a usability questionnaire was used to collect user feedback on the ease of use and enjoyment. The system conceptualization originated from the transformation of traditional rehabilitation methods into electronic formats, aiming to alleviate the workload of health care professionals, enhance the precision of rehabilitation prescriptions, digitize records, and introduce an element of entertainment into patient treatment. We commenced extensive testing with a large number of participants to ascertain the clinical effectiveness of the system.

#### Operating Modes and Time Settings

All participants were asked to use both BP and CE rehabilitation modes. The practice sessions involved completing 15 basic shape pairings within 30 minutes and 15 shape and color recognitions within 40 minutes. The completion rates for each session were examined to validate the appropriateness of the time settings in the prototype.

#### Correlation Between Manual Dexterity and Cognitive Function

To investigate whether the proposed pegboard can enhance both manual dexterity and cognitive function, all participants underwent initial measurements of grip-and-pinch strength. Following this, assessments of manual dexterity were conducted using standardized tools, such as the Box-and-Block test (BBT). Subsequently, participants underwent cognitive assessments, including the Trail-Making Test (TMT) and the Mini-Mental Status Examination (MMSE). This sequential process was designed to examine the correlation between the use of the proposed pegboard and the enhancement of both manual dexterity and cognitive abilities.

#### Usability Assessment

To comprehensively assess user interactions with the proposed system, this study used the System Usability Scale (SUS) [28]. Administered through a 5-point Likert scale, the SUS probed user perceptions of the pegboard’s difficulty and user acceptance of the system in order to understand whether the time and task settings were appropriate. The insights gained from user experiences will serve as valuable references for enhancing and refining the prototype.

### Data Analysis

This study used clinical assessments conducted by physicians to evaluate participants’ physical and mental conditions, testing conditions, and experimental procedures to recommend their participation in prototype testing. Throughout the testing phase, there were no missing data or participant withdrawals. Questionnaires were administered sequentially, with responses entered without any transformations into SPSS version 20.0 for statistical analysis.

This study used SPSS version 20.0 for all statistical analyses. Data analysis focused on examining differences in operating modes, time settings, and difficulty levels in comparison to patients’ capabilities. Furthermore, to explore potential associations between manual dexterity and cognitive faculties among patients with stroke, we used the Spearman rank correlation coefficient. Pearson correlation coefficients were used to assess the correlation between BP-1 completion rates and scores of BP-2, CE-1, and CE-2 pertaining to accuracy/completion rates. This analysis aimed to investigate the relationship between operational proficiency and cognitive abilities. *P*<.05 was set as the measure of statistical significance. This study also analyzed user experiences to assess the feasibility of digitized manual dexterity and cognitive function training. These analytical approaches enhanced the scientific rigor of our findings and provided a nuanced understanding of the interrelationships among the variables under investigation.

## Results

### Demographic Data

The gender and age distributions of the 10 participants in the study are presented in Table 1.

**Table 1:** Demographic data of participants (N=10).

Variable		Participants, n (%)
**Gender**		
	Male	6 (60)
	Female	4 (40)
**Age (years)**		
	55-65	3 (30)
	66-75	4 (40)
	76-85	2 (20)
	≥86	1 (10)

### Interactive Electronic Pegboard Versus Neofect Smart Pegboard

The interactive electronic pegboard developed in this study not only incorporates all the features of the Neofect Smart Pegboard but also allows patients to record their outcomes in the cloud after each practice session through software and hardware integration. The accumulation of data then enables the system to tailor rehabilitation courses to individual patient needs. The proposed interactive electronic pegboard also offers greater flexibility in gamification, as users can expand their rehabilitation options by simply purchasing additional game modules. The system’s distinctiveness and progression are illustrated in Table 2.

**Table 2:** Comparative analysis of the Neofect Smart Pegboard vs the interactive electronic pegboard.

Category	Neofect Smart Pegboard	Interactive electronic pegboard
Objectives	Provides analysis manual dexterity, visual motor integration, visual cognition, and shape recognition	Provides analysis for manual dexterity, hand-eye coordination, visual cognition, and shape recognition
Dimensions	557 (width) × 353 (height) × 32 (depth) mm^3^	258 (width) × 163 (height) × 7.5 (depth) mm^3^
Weight	6.32 kg	490 g
Sensor	Hole sensor (magnetic recognition)	Capacitive displacement sensor
Speaker	Built-in speaker for auditory feedback	Built-in speaker for auditory feedback
Digital training program	Delivers outcome-driven digital rehabilitation training targeting upper limb, visual/spatial, and cognitive capabilities	Provides outcome-driven digital rehabilitation training tailored to enhance upper limb, visual/spatial, and cognitive abilities
Visual feedback	Featuring high-intensity light-emitting diodes, offers visual cues to guide the placement of pegs	Using the tablet screen, provides prompts to guide users in placing building blocks in correct positions
Auditory feedback	Enhances the training experience by using voice and sound effects for patient guidance	Provides patients with prompts by incorporating built-in voice and musical effects on the tablet, indicating the correctness of their responses and thereby enhancing the training experience
Various peg sets	By effortlessly substituting pegboards, individuals can access diverse rehabilitation training tailored to their specific training objectives.	Using the system’s diverse gaming features, patients can enhance rehabilitation training versatility by easily replacing the tablet’s outer casing, thereby adapting the training pegboard to their individual rehabilitation needs.
Training result	Presents real-time test results, including the total time, number of successful pegs, average peg movement time, and success rate	Provides a real-time display of test results, encompassing the user goal achievement rate, number of successful pegs, analysis of reasons for unmet goals (related to motion or cognitive issues), success rate, and goal predictions
Session training	Users can customize training sessions by selecting specific exercises to suit their needs and follow the tailored session accordingly.	Therapists can preset relevant exercises or customize training programs based on user needs, adjusting them according to the observed outcomes.
Wide range of difficulty levels	Can configure various difficulty levels for the game and adjust settings, such as the time, speed, and number of blocks	Can adjust game difficulty and modify settings, such as the time, speed, and number of blocks, and supports both offline and online practice, featuring online question-and-answer functionalities

### Test Results of Operating Modes and Time Settings

The findings indicated that even when using their dominant right hand, the vast majority of participants encountered difficulties in successfully completing tasks within the allocated time frames of the prototype (30 minutes for BP and 40 minutes for CEs). Such observations indicated that when using the proposed system to complete rehabilitation tasks, participants encountered certain limitations.

The experiment was broadly divided into 2 phases. In BP-1 (motor training), the highest score was 12 out of 15. However, upon transitioning to BP-2 and with the introduction of color variables, some participants experienced a significant decline in operating speed and accuracy, although the highest score of 12 was maintained. In BP-2, similar to BP-1, participants were required to complete 15 block pairings within 30 minutes in CE-1 and 15 pairings with color and shape variables within 40 minutes in CE-2. The best performance score in CE-1 was 11 out of 15. However, the introduction of color variables in CE-2 meant participants were unable to complete tasks within the designated time, with a corresponding rise in error rates; the highest score remained 11 out of 15. The test results of difficulty and time settings from BP-1 to CE-2 indicated a gap between the current time settings and the capabilities of patients (Table 3).

**Table 3:** User performance (scores out of 15).

Case	BP^a^-1^b^ score, n (%)	BP-2^c^ score, n (%)	CE^d^-1^b^ score, n (%)	CE-2^c^ score, n (%)
1	8 (53.3)	8 (53.3)	11 (73.3)	7 (46.7)
2	12 (80.0)	11 (73.3)	11 (73.3)	9 (60.0)
3	12 (80.0)	10 (66.7)	11 (73.3)	11 (73.3)
4	10 (66.7)	10 (66.7)	11 (73.3)	9 (60.0)
5	11 (73.3)	10 (66.7)	11 (73.3)	8 (53.3)
6	12 (80.0)	12 (80.0)	11 (73.3)	11 (73.3)
7	9 (60.0)	8 (53.3)	9 (60.0)	8 (53.3)
8	9 (60.0)	9 (60.0)	8 (53.3)	8 (53.3)
9	8 (53.3)	7 (46.7)	7 (46.7)	7 (46.7)
10	10 (66.7)	9 (60.0)	10 (66.7)	9 (60.0)

^a^BP: basic practice.

^b^Excluding color recognition variations.

^c^Introducing color perception variables.

^d^CE: cognitive exercise.

### Relationship Between System Scores and Functional Assessments

The experimental results indicated a negative correlation between participants’ performance in BP-1 and CE-1 and the BBT, suggesting that with longer training durations, the effectiveness of training for manual dexterity and coordination abilities in patients’ hands decreased. Furthermore, after multiple sessions of color and shape cognition exercises and tests in BP-2 and CE-2, there were significant changes in participants’ performance in the TMT, confirming that the cognitive training was helpful to the participants. Finally, as illustrated in Table 4, among the 10 participants, there was a significant correlation between the MMSE scores and color and shape cognition scores of the BP-2 and CE-2.

**Table 4:** Correlations between system scores and dexterity/cognitive evaluations.

Evaluation	BBT^a^	TMT^b^	MMSE^c^
BP^d^-1	–0.63^e^	0.21	0.32
BP-2	–0.58^e^	0.41^f^	0.52^f^
CE^g^-1	–0.72^e^	0.29	0.37
CE-2	–0.41	0.53^e^	0.61^e^

^a^BBT: Box-and-Block test.

^b^TMT: Trail-Making Test.

^c^MMSE: Mini-Mental State Examination.

^d^BP: basic practice.

^e^Spearman rank correlation analysis (N=10); *P*<.01.

^f^Spearman rank correlation analysis (N=10); *P*<.05.

^g^CE: cognitive exercise.

In this study, Pearson correlation coefficients (r) were used to assess the potential association between patients’ operational proficiency (BP-1) and cognitive abilities (BP-2, CE-1, CE-2). The correlations for each domain were as follows: BP-2 (r=0.911, *P*<.001), CE-1 (r=0.653, *P*=.04), and CE-2 (r=0.852, *P*=.002). For BP-2 and CE-2, the correlation was significant at 0.01, while for CE-1, it was significant at 0.05.

In summary, a series of test results indicated that as patients engage in cognitive tasks, their operational capacity tends to decline with increasing cognitive load. As evidenced by the experiments, based on BP-1 (with no color or shape recognition variables), participants’ performance slightly deteriorated during the CE-1 test involving shape recognition. Furthermore, upon progressing to BP-2 (introducing color variables), participants’ response efficiency diminished compared to BP-1. Subsequently, in CE-2 (incorporating color and shape recognition variables), a significant disparity in overall performance became evident, thus confirming a notable correlation between operational capacity and cognitive load.

### System Usability

**Figure 5:**
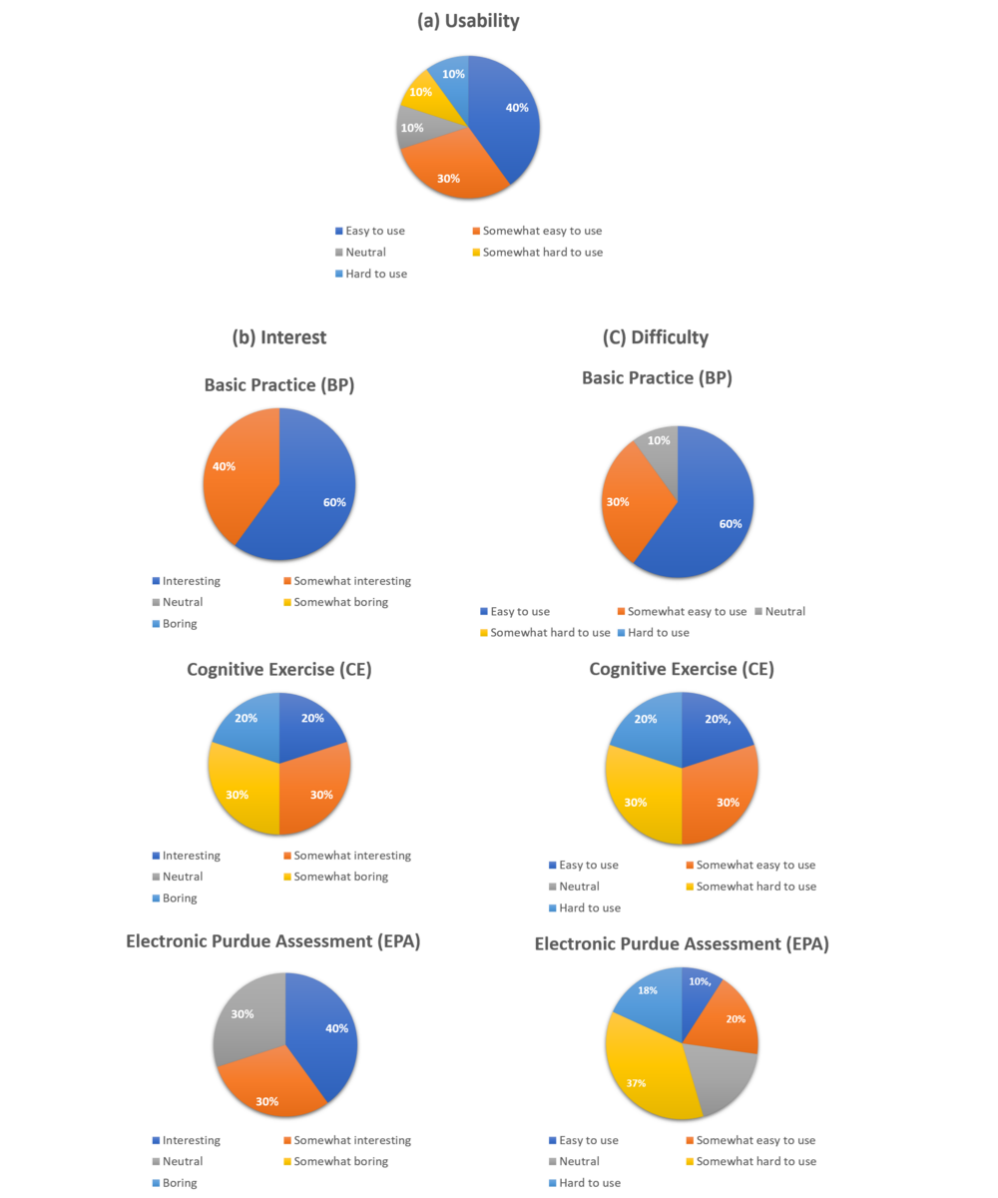
Results of (a) system design usability testing, (b) user acceptance testing, and (c) operational difficulty testing.

To develop a prototype of the pegboard for functional and task assessments, questionnaire interviews were conducted simultaneously to collect user feedback for this study. In the usability assessment, approximately 70% (n=7) of the participants reported that the proposed system is notably user friendly (Figure 5a). A significant consensus was also observed among participants regarding the transition from traditional building blocks to electronic blocks during BP training, with nearly 70% (n=7) finding that the proposed system is engaging. Note that when participants entered the next stage of CE training, only 50% (n=5) of them considered the platform to be interesting (Figure 5b). Finally, in the evaluation of task difficulty, the majority of participants (n=9, 90%) perceived the BP training tasks as easy and simple. However, upon advancing to the more complex CE training, which involved increased shape and color recognition, a noticeable increase in perceived task complexity was observed. Only 20% (n=2) of the participants regarded this phase as easy, while the vast majority found it somewhat difficult and challenging (Figure 5c).

## Discussion

### Principal Findings

#### Prototype Setup and Testing

In the preliminary investigation, the preset time frames on the prototype did not align with the capabilities of participants, and none of the participants were able to complete the tasks within the designated time, which could be attributed to a decline in hand/finger muscle strength, manipulation, walking ability, and information processing speed associated with aging, as documented in previous relevant studies. These findings emphasize the importance of considering age-related factors in the design and calibration of such interactive systems [19,20,28]. Additionally, due, perhaps, to the lack of electronic records, therapists tended to focus more on how many blocks users can complete, rather than paying attention to how far users are from the goal. Therefore, digitized rehabilitation could help clarify a patient’s recovery status. Moreover, in both BP-1 and CE-1 modes, the electronic block exercises exhibited a negative correlation with improvement in motor skills. This could be attributed to factors such as time settings or user habits. At the same time, aging could contribute to a decline in dexterity and attention [29,30]. Heintz Walters et al [31] applied eye-tracking metrics during commonly used dexterity tests and found that older adults exhibit poorer visual focus and reaction capabilities. Therefore, in future studies, we plan to explore the relationship between eye movements and performance in each of the system tasks for both healthy participants and patients with stroke [32-35].

#### Relationship Between System Scores and Functional Assessments

Although the results from BP-1 and CE-1 indicate a significant negative correlation between digital block exercises and manual dexterity, surprising outcomes emerged in subsequent BP-2 and CE-2 training sessions, when variations in color and shape were introduced. The majority of participants exhibited a notable correlation between performance levels on the TMT and the MMSE. This implies a correlation between patients’ operational capabilities and cognition. In the past, discerning whether difficulties faced by patients in completing motor tasks stem from operational capabilities or cognitive issues with traditional rehabilitation aids has been challenging. However, our system not only records patient completion rates but also conducts an analysis of color accuracy. By doing so, the system not only assesses patients’ operational abilities but also provides an initial clarification of their cognitive status. These unexpected findings suggest that the increased cognitive challenges posed by engaging in exercises with diverse shapes and colors could stimulate and potentially influence patients’ manual dexterity and attention-switching abilities, consequently enhancing their cognitive functions. Moreover, this digitized tool, enhanced with guiding indicator lights, not only facilitates precise block pairings but also is a critical element in enhancing users’ visual perception and attention [36-38]. Simultaneously, the incorporation of sound and musical guidance can further enhance the training’s engaging nature.

#### Evaluation of Usability, Appeal, and Difficulty

The majority of participants perceived the proposed interactive electronic pegboard as highly user friendly and easy to operate. This can be attributed to the size of the prototype, which closely resembles that of traditional training pegboards, minimizing the degree of adaptation needed. Moreover, the design of the pegs adheres to common training block proportions, which are crafted in a 1:1 ratio to ensure they are easy to grasp—not too large and not too small. In addition, a significant advancement in this design lies in the elimination of manual recording by therapists, along with the need for separate stopwatches. This represents notable progress, as traditional rehabilitation sessions of this nature often require 1-on-1 interaction with health care professionals. This is not only labor intensive but also error prone.

In this preliminary trial, more than two-thirds (70%) of the participants considered BP training to be simple, and almost 60% of the participants found this digital tool to be interesting. However, as operating constraints increased (considering both shape and color elements simultaneously), most participants reported that the tasks became more challenging. Flexibility is a major advantage of this tool as it is easy to adjust both the difficulty level and the richness of training. This feature is particularly crucial for patients requiring long-term rehabilitation. Taking patients with stroke as an example, not only does prolonged rehabilitation imposes physical strain, but the repetitive nature of exercises without feedback can also lead to a lack of motivation and fatigue among patients. Gamification of rehabilitation can assist in alleviating the monotony associated with extended rehabilitation sessions by incorporating game-like variations. Simultaneously, for patients with stroke requiring long-term rehabilitation, maintaining rehabilitation efforts at home postdischarge is a critical determinant of recovery effectiveness. In contrast to conventional rehabilitation approaches, this electronic rehabilitation assistive equipment demonstrates notable progress in terms of labor costs, rehabilitation documentation, medical precision, and the gamification of rehabilitation, making it a promising trend in future health care. In the future, we plan to connect the device to multiuser platforms so that a competitive dimension can be introduced to stimulate engagement [39-41]. In this initial experimental trial, none of the participants were able to complete the tasks within the designated time, suggesting that there is room for improvement in terms of the time settings of the prototype. Simultaneously, concerning clinical needs, rehabilitation records serve as crucial reference indicators for the formulation of rehabilitation programs (prescriptions). To further enhance this system as a rehabilitation aid, the system has initiated the design of graphical interfaces to visualize the rehabilitation process and progress. This facilitates health care professionals in promptly understanding the improvement status of patients’ hand functionality. In the next phase of study, we plan to focus on refining and enhancing these aspects [42,43].

### Conclusion

This study aimed at validating product functionality and a concept, and preliminary testing was conducted exclusively in the rehabilitation outpatient clinic of a northern teaching hospital. Although the findings confirm the feasibility of the concept, the limited sample size prevents the extrapolation of results to other health care institutions. Therefore, larger-scale clinical trials will be implemented once the system is fully optimized. Moreover, the study did not evaluate additional functions of the prototype (eg, the digitalized PPT), primarily due to the excessive sensitivity of the sensor components at this stage, along with time and technological constraints. These functionalities will be subjected to further testing and comparative analysis following future system refinements, thereby enhancing the comprehensiveness and robustness of the research findings.

In this investigation, we developed a novel interactive electronic pegboard—a comprehensive software and hardware system specifically crafted for rehabilitation of patients with stroke. This system was used to evaluate dexterity and cognitive functions through 3 task types and multiple demonstration patterns.

We recruited patients with stroke who had previously used conventional rehabilitation methods and asked them to perform preliminary tests on the proposed system. Through meticulous data analysis, we assessed the hand dexterity and cognitive functional abilities of the patients. The initial test results indicate that the proposed system is effective in terms of both rehabilitation and assessment. In the future, following further enhancement and stabilization of the system, we plan to conduct extensive usability testing with a large user (patient) population. The testing protocol will encompass user interactions, message comprehension, and interface operability, among other aspects. Additionally, we will use the System Usability Scale (SUS) questionnaire to validate the clinical feasibility of the product. Furthermore, to ensure the reliability and validity of future product applications in clinical settings, the experiment will cautiously assess the linear correlation of variance during large-scale trials and use power analysis to analyze the significance level, power, and effect size. This is to ensure the rationality of the clinical statistical sample size and enhance the reliability of the statistical analysis. The primary objective of this research was to not only bridge the gap between clinical needs and product development but also go beyond commercially available products by enabling tracking of individual rehabilitation progress [44-47]. This tracking enables the development of personalized rehabilitation programs based on individual differences [48-51]. Ultimately, our goal was to develop an intelligent system capable of delivering user-friendly optimized rehabilitation regimens to meet the diverse needs of users and substantively contribute to the benefits brought about by clinical validation.

## Abbreviations

BBT: Box-and-Block test
BP: basic practice
CE: cognitive exercise
EPA: Electronic Purdue Assessment
MMSE: Mini-Mental Status Examination
PPT: Purdue Pegboard Test
TMT: Trail-Making Test

## References

[1] Blume SW, Curtis JR (2011). Medical costs of osteoporosis in the elderly Medicare population. Osteoporos Int.

[2] He W, Goodkind D, Kowal P (2016). An aging world: 2015. International population reports. US Census Bureau.

[3] Fineman MA (2012). 'Elderly' as vulnerable: rethinking the nature of individual and societal responsibility. SSRN J.

[4] Pearlin L (1999). The stress process revisited: Reflections on concepts and their interrelationships. Handbook of the Sociology of Mental Health.

[5] Conger RD, Ge X, Elder GH, Lorenz FO, Simons RL (1994). Economic stress, coercive family process, and developmental problems of adolescents. Child Dev.

[6] MacMahon S, Peto R, Cutler J, Collins R, Sorlie P, Neaton J, Abbott R, Godwin J, Dyer A, Stamler J (1990). Blood pressure, stroke, and coronary heart disease. Part 1, prolonged differences in blood pressure: prospective observational studies corrected for the regression dilution bias. Lancet.

[7] Gorelick PB, Scuteri A, Black SE, Decarli C, Greenberg SM, Iadecola C, Launer LJ, Laurent S, Lopez OL, Nyenhuis D, Petersen RC, Schneider JA, Tzourio C, Arnett DK, Bennett DA, Chui HC, Higashida RT, Lindquist R, Nilsson PM, Roman GC, Sellke FW, Seshadri S, American Heart Association Stroke Council‚ Council on EpidemiologyPrevention‚ Council on Cardiovascular Nursing‚ Council on Cardiovascular RadiologyIntervention‚Council on Cardiovascular SurgeryAnesthesia (2011). Vascular contributions to cognitive impairment and dementia: a statement for healthcare professionals from the american heart association/american stroke association. Stroke.

[8] Manji RA, Arora RC, Singal RK, Hiebert BM, Menkis AH (2017). Early rehospitalization after prolonged intensive care unit stay post cardiac surgery: outcomes and modifiable risk factors. JAHA.

[9] Filsoufi F, Rahmanian PB, Castillo JG, Bronster D, Adams DH (2008). Incidence, topography, predictors and long-term survival after stroke in patients undergoing coronary artery bypass grafting. Ann Thorac Surg.

[10] Apfelbaum JL, Chen C, Mehta SS, Gan ATJ (2003). Postoperative pain experience: results from a national survey suggest postoperative pain continues to be undermanaged. Anesth Analg.

[11] Kwakkel G, Kollen B, Lindeman E (2004). Understanding the pattern of functional recovery after stroke: facts and theories. Restor Neurol Neurosci.

[12] Mahncke HW, Bronstone A, Merzenich MM (2006). Brain plasticity and functional losses in the aged: scientific bases for a novel intervention. Progress Brain Res.

[13] Moriarty O, McGuire BE, Finn DP (2011). The effect of pain on cognitive function: a review of clinical and preclinical research. Prog Neurobiol.

[14] Hertzog C, Kramer AF, Wilson RS, Lindenberger U (2008). Enrichment effects on adult cognitive development: can the functional capacity of older adults be preserved and enhanced?. Psychol Sci Public Interest.

[15] Duncan PW, Zorowitz R, Bates B, Choi JY, Glasberg JJ, Graham GD, Katz RC, Lamberty K, Reker D (2005). Management of adult stroke rehabilitation care. Stroke.

[16] Quinn T, Paolucci S, Sunnerhagen K, Sivenius J, Walker M, Toni D, Lees K, European Stroke Organisation (ESO) Executive Committee (2009). Evidence-based stroke r-ehabilitation: an expanded guidance document from the European Stroke Organisation (ESO) guidelines for management of ischaemic stroke and transient ischaemic attack 2008. J Rehabil Med.

[17] Heinemann AW, Linacre JM, Wright BD, Hamilton BB, Granger C (1993). Relationships between impairment and physical disability as measured by the functional independence measure. Arch Phys Med Rehabil.

[18] Majmudar S, Wu J, Paganoni S (2014). Rehabilitation in amyotrophic lateral sclerosis: why it matters. Muscle Nerve.

[19] Sawyer D, CDRH Work Group (1996). An introduction to human factors in medical devices. US Department of Health and Human Services, Public Health Service, Food and Drug Administration, Center for Devices and Radiological Health.

[20] Gabriels RL, Agnew JA, Holt KD, Shoffner A, Zhaoxing P, Ruzzano S, Clayton GH, Mesibov G (2012). Pilot study measuring the effects of therapeutic horseback riding on school-age children and adolescents with autism spectrum disorders. Res Autism Spectr Disord.

[21] Proud E, Bilney B, Miller KJ, Morris ME, McGinley JL (2019). Measuring hand dexterity in people with Parkinson’s disease: reliability of pegboard tests. Am J Occup Ther.

[22] Jaeger J (2018). Digit symbol substitution test: the case for sensitivity over specificity in neuropsychological testing. J Clin Psychopharmacol.

[23] Milberg WP, Hebben N, Kaplan E, Grant I, Adams KM (2009). The Boston Process Approach to neuropsychological assessment. Neuropsychological Assessment of Neuropsychiatric and Neuromedical Disorders.

[24] Bontis N, Fitz-enz J (2002). Intellectual capital ROI: a causal map of human capital antecedents and consequents. J Intellect Cap.

[25] Dao V, Langella I, Carbo J (2011). From green to sustainability: information technology and an integrated sustainability framework. J Strateg Inf Syst.

[26] Rahmani AM, Gia TN, Negash B, Anzanpour A, Azimi I, Jiang M, Liljeberg P (2018). Exploiting smart e-Health gateways at the edge of healthcare internet-of-things: a fog computing approach. Future Gener Comput Syst.

[27] Chekroud AM, Bondar J, Delgadillo J, Doherty G, Wasil A, Fokkema M, Cohen Z, Belgrave D, DeRubeis R, Iniesta R, Dwyer D, Choi K (2021). The promise of machine learning in predicting treatment outcomes in psychiatry. World Psychiatry.

[28] Proud EL (2016). Manual dexterity evaluation in people with Parkinson’s disease. University of Melbourne.

[29] Rikli RE, Jones CJ (1999). Functional fitness normative scores for community-residing older adults, ages 60-94. JAPA.

[30] Keogh J, Kilding A, Pidgeon P, Ashley L, Gillis D (2009). Physical benefits of dancing for healthy older adults: a review. J Aging Phys Act.

[31] Heintz Walters B, Huddleston WE, O'Connor K, Wang J, Hoeger Bement M, Keenan KG (2021). The role of eye movements, attention, and hand movements on age-related differences in pegboard tests. J Neurophysiol.

[32] Miller EL, Murray L, Richards L, Zorowitz RD, Bakas T, Clark P, Billinger SA (2010). Comprehensive overview of nursing and interdisciplinary rehabilitation care of the stroke patient: a scientific statement from the American Heart Association. Stroke.

[33] Knopman D, Boeve BF, Petersen RC (2003). Essentials of the proper diagnoses of mild cognitive impairment, dementia, and major subtypes of dementia. Mayo Clin Proc.

[34] Beck AT (1979). Cognitive Therapy of Depression.

[35] Young JE, Weinberger AD, Beck AT, Barlow DH (2001). Cognitive therapy for depression. Clinical Handbook of Psychological Disorders: A Step-by-Step Treatment Manual (3rd ed.).

[36] Wheeler ME, Treisman AM (2002). Binding in short-term visual memory. J Exp Psychol Gen.

[37] Luria R, Vogel EK (2011). Shape and color conjunction stimuli are represented as bound objects in visual working memory. Neuropsychologia.

[38] Luria R, Vogel EK (2011). Visual search demands dictate reliance on working memory storage. J Neurosci.

[39] Laver K (2020). Virtual reality for stroke rehabilitation. Virtual Reality in Health and Rehabilitation.

[40] Van Den Berg AE, Hartig T, Staats H (2007). Preference for nature in urbanized societies: stress, restoration, and the pursuit of sustainability. J Soc Issues.

[41] Ulrich RS, Simons RF, Losito BD, Fiorito E, Miles MA, Zelson M (1991). Stress recovery during exposure to natural and urban environments. J Environ Psychol.

[42] Acharya KA, Bhat S, Kanthi M, Rao B (2022). Fine motor assessment in upper extremity using custom-made electronic pegboard test. J Med Signals Sens.

[43] Al-Naami B, Al-Naimat F, Almalty ARM, Visconti P, Al-Hinnawi A (2021). A prototype of an electronic pegboard test to measure hand-time dexterity with impaired hand functionality. ASI.

[44] Weintraub S, Salmon D, Mercaldo N, Ferris S, Graff-Radford NR, Chui H, Cummings J, DeCarli C, Foster NL, Galasko D, Peskind E, Dietrich W, Beekly DL, Kukull WA, Morris JC (2009). The Alzheimer's Disease Centers' Uniform Data Set (UDS): the neuropsychologic test battery. Alzheimer Dis Assoc Disord.

[45] Taylor J, Taylor S (1997). Psychological Approaches to Sports Injury Rehabilitation.

[46] Sohlberg MM, Mateer CA (2001). Cognitive Rehabilitation: An Integrative Neuropsychological Approach.

[47] Borghese NA, Pirovano M, Lanzi PL, Wüest S, de Bruin ED (2013). Computational intelligence and game design for effective at-home stroke rehabilitation. Games Health J.

[48] Bush DEA, Sotres-Bayon F, LeDoux JE (2007). Individual differences in fear: isolating fear reactivity and fear recovery phenotypes. J Trauma Stress.

[49] Sung M, Marci C, Pentland A (2005). Wearable feedback systems for rehabilitation. J Neuroeng Rehabil.

[50] Sveistrup H (2004). Motor rehabilitation using virtual reality. J Neuroeng Rehabil.

[51] Novak D (2018). Promoting motivation during robot-assisted rehabilitation. Rehabilitation Robotics.

